# Knowledge and Beliefs of EMS Providers toward Lights and Siren Transportation

**DOI:** 10.5811/westjem.2015.2.24212

**Published:** 2015-04-06

**Authors:** Joseph Tennyson, Louise Maranda, Adam Darnobid

**Affiliations:** * University of Massachusetts Medical School, Worcester, Massachusetts; † University of Massachusetts Medical School, Department of Quantitative Health Sciences and Pediatrics, Worcester, Massachusetts; ‡ UMass Memorial Health Alliance Hospital, Leominster, Massachusetts

## Abstract

**Introduction:**

The use of warning lights and siren (WLS) increases the risk of ambulance collisions. Multiple studies have failed to demonstrate a clinical benefit to the patients. We sought to investigate the degree to which providers understand the data and incorporate it into their practice.

**Methods:**

The authors distributed an anonymous survey to prehospital providers under their medical direction at staff and quality assurance meetings. The surveys asked the providers’ degree of agreement with four statements: transport with lights and siren shortens transport times; transport with lights and siren improves patient outcome; transport with lights and siren increases the risk of collision during transport; and transport with lights and siren reduces the utilization of “mutual aid” service. We compared responses between providers who had been in prior ambulance collisions and those who had not.

**Results:**

Few responses reached statistical significance, but respondents tended towards agreement that WLS use shortens transport times, that it does not improve outcomes, and that it increases the risk of collision. Despite the overall agreement with the published literature, respondents report >80% of transports are conducted using WLS.

**Conclusion:**

The data demonstrate the surveyed providers are aware of the risk posed by WLS to themselves, their patients, and the public. Nevertheless, their practice in the absence of rigid protocols suggests they disregard this knowledge. Despite a large number of prior ambulance collisions among the surveyed group, a high number of transports are conducted using WLS.

## INTRODUCTION

Ambulance collisions represent a risk for the emergency medical services (EMS) providers who operate on the front lines of our healthcare system.^1^–^9^ EMS personnel in the United States have more than twice the annual occupational fatality rate of the general public.^2^ Many of these fatalities occur during the operation of ambulances.^2^,^9^ Operation of the ambulance with warning lights and siren (WLS) is associated with an increased rate of collisions.^3^,^4^,^6^,^8^ These collisions cause a loss of both life and resources. Further, there is a demonstrated increase in the risk of personal injury and death in collisions that occur under WLS operation.^4^,^6^,^8^ Research has shown that time saved in using WLS for patient transports ranges from less than one minute to almost four minutes.^10^–^14^ Research evaluating the clinical benefit of use of WLS has shown a small benefit of decreased field times in penetrating trauma,^15^ but the remainder of the literature examined is negative.^16^–^19^ The National Association of EMS Physicians (NAEMSP) has issued a position statement calling for limitation of the use of WLS to “emergency situations only.”^20^ Because there is no clear definition of an emergency situation, practices vary tremendously from service to service.

Literature has suggested that field providers are aware of the increased risk borne in operating with WLS.^21^ The authors’ personal observations of practice in our region reveal that many services continue to routinely use WLS for the transport phase of 911 calls. It is also unclear why providers do not incorporate the knowledge of increased risk and minimal benefit into their practice. We designed this study to evaluate the field level providers’ awareness of the potential problem. Based on our observations of the practice of providers in our region, it is our hypothesis that field providers do not understand the risk associated with WLS and that they believe it improves outcomes and system performance. We further hypothesized that those providers who had experienced ambulance collisions personally would have a greater understanding of the risk, the marginal time benefit, and the lack of proven clinical benefit.

## METHODS

### Participants

We distributed the survey at staff and quality assurance meetings. Participants represented a diverse sample of prehospital providers under the medical direction of EMS physicians from the authors’ group. The participants included practicing field emergency medical technicians (EMTs) and paramedics from fire-based EMS, hospital-based EMS, and private companies providing both emergency response and transfer services. The providers surveyed represented a geographical distribution including suburban and urban environments. The services surveyed had annual 911 call volumes ranging from 1,100 to over 30,000. Because many providers work for multiple services across the above domains, it was impractical to stratify responses by type of employment. At the time that this survey was conducted, there existed no generalized protocol towards the use of lights and siren. Individual services generally left the decision regarding their use to the individual provider.

### Study Design

The local institutional review board waived full review for this observational, anonymous survey-based study of both Advanced Life Support (ALS) and Basic Life Support (BLS) providers. The demographics weobtained included age, gender, level of certification (ALS or BLS), number of years in service, and number of accidents. The respondents were also asked for an estimate of the percentage of their own transports that were conducted using WLS. This estimate was not stratified by transfer or emergency response role.

We surveyed participants using a 10-point scale, assessing the degree to which the provider agrees with the following statements (1 equals “Not at all”, 4–5 equals “Unsure”, 10 equals “Strongly Agree”):

1. Transport with lights and siren shortens transport times.
2. Transport with lights and siren improves patient outcome.
3. Transport with lights and siren increases the risk of collision during transport.
4. Transport with lights and siren reduces the utilization of “mutual aid” service.

### Statistical Analyses

We performed overall comparisons of the distribution of responses using the Kolmogorov–Smirnov test. The comparison of median response frequencies were done using the Mann-Whitney U test. Analyses were done with SPSS, version 21 (Armonk, NY). We prepared histograms of total response by category using Microsoft Excel version 14.0. Trendlines were applied and displayed with R^2^ values to aid in visual interpretation of trends.

## RESULTS

**Figure 1:**
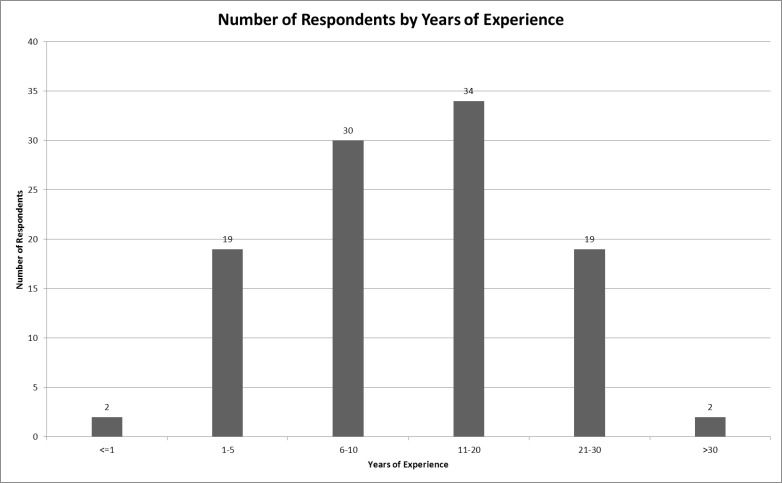
Experience of respondents in years.

The response rate was 100% for the 108 surveys distributed. All 108 surveys returned contained responses to the primary survey questions. Because some surveys were incomplete in the areas of demographics and background information, we performed analysis based on the data available for each individual response. Specifically, four surveys did not include the respondents’ age, two did not include the extent of their experience, and one survey did not include gender. Table 1 shows the overall characteristics of respondents. The mean age was 35 and the mean total experience level was 13 years. Figure 1 shows the distribution of total years of experience of respondents. Respondents’ estimation of the percentage of their transports conducted using WLS revealed that approximately 82% of transports were conducted in this manner. ALS providers estimated 89% WLS transports vs. 61% for BLS providers (p<0.001).

**Figure 2:**
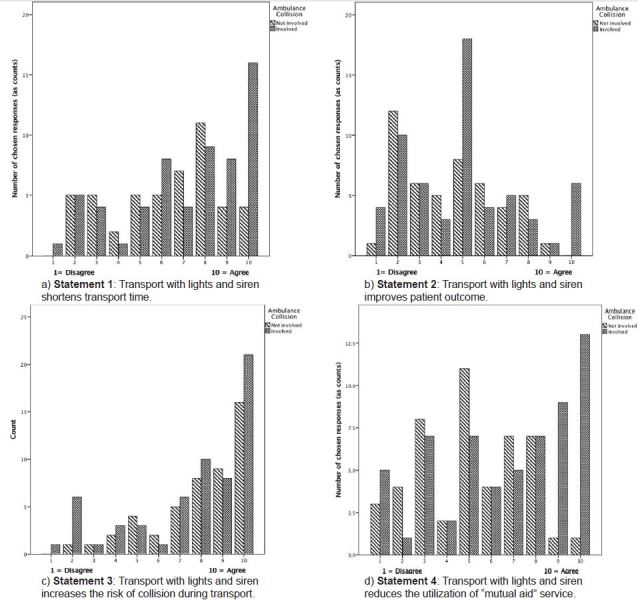
Distribution of responses from emergency medical services providers separated by whether they had experienced a prior ambulance collision.

**Figure 3:**
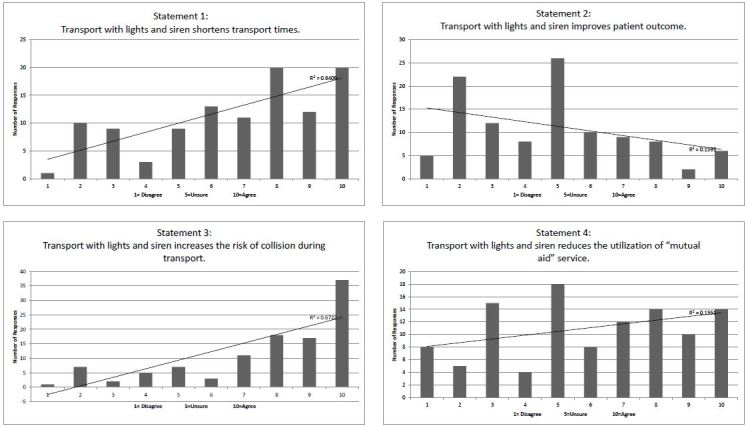
Distribution of total responses from emergency medical services (EMS) providers.

Respondents reported 147 collisions (Table 2). One provider reported 12 collisions. Respondents reported a cumulative total of 1,380 years of experience yielding a rate of 0.1 collisions per EMS year of service, or onecollision for every 10 providers each year. Forty percent of these collisions were reported as occurring during WLS operation. Figure 2 shows the responses of providers separated by whether they had previously experienced and ambulance collision. Figure 3 provides histograms of the total responses to each statement.

### Statement 1

Transport with lights and siren shortens transport times.

We found a difference in the distribution of answers from those involved in an accident, compared to those not involved in an accident, which approaches but does not achieve significance (p=0.110).

Comparing median responses did not yield a significant difference (p=0.162), which can be confirmed visually for almost all categories of responses.

### Statement 2

Transport with lights and siren improves patient outcome.

We did not find a significant difference between overall answers from those involved in an accident, compared to those not involved in an accident (p=0.861).

Comparing median responses did not yield a significant difference (p=0.982), which can be confirmed visually for almost all categories of responses.

### Statement 3

Transport with lights and siren increases the risk of collision during transport.

We did not find a significant difference between overall answers from those involved in an accident, compared to those not involved in an accident (p=0.952).

Again, comparing median responses did not yield a significant difference (p=0.846), which can be confirmed visually for all categories of responses.

### Statement 4

Transport with lights and siren reduces the utilization of “mutual aid” service.

We found a significant difference between overall answers from those involved in an accident, compared to those not involved in an accident (p=0.007). Comparing median responses yielded a significant difference (p=0.003), which can be confirmed visually for the most extreme categories of agreement responses.

**Figure 4:**
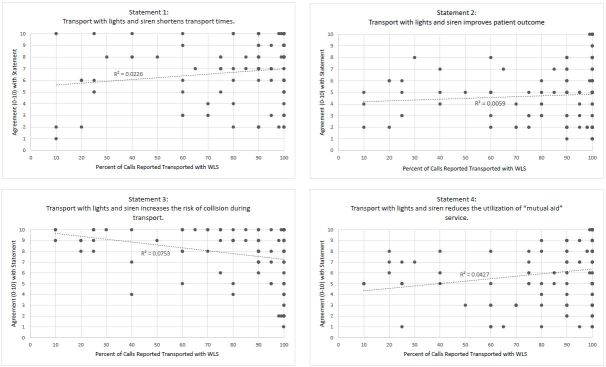
Comparison of statement responses with providers’ reported warning lights and siren (WLS) use.

Individual respondents’ estimated percentage of transports with WLS was compared to their responses into the survey questions in Figure 4. Scatter plots with R^2^ values show a lack of correlation between the response and the percentage of WLS transports for any of the survey questions.

## DISCUSSION

Among surveyed EMS providers, a knowledge of the lack of clear benefit and the increased risk of WLS use is not associated with a reduction in the use of WLS by the surveyed providers. Despite a trend toward agreement with the concept that WLS increased the risk of collisions, greater than 80% of transports in our surveyed group were transported using WLS. The fact that a provider had a prior ambulance collision did not significantly influence the providers’ belief in the risk of using WLS. Few prior works have addressed the knowledge base and beliefs of prehospital providers toward the published data on risks associated with WLS. One recent paper demonstrated providers’ concern for these risks and their concern that too many protocols required WLS response.^21^ This study, conducted in another state, suggests that there is a developing concern for the risks associated with this practice and that the practice patterns revealed in this survey may be a regional cultural phenomenon.

Considerable evidence consistently reported over the years has associated the use of WLS with an increased risk of collision, injury and fatality.^2^–^8^ The responses suggest that the providers surveyed are aware of this risk. Despite this, survey respondents estimated that more than 80% of transports were conducted using WLS. At the time of the survey, the region in which the surveyed providers practice had no specific protocols regarding the use of WLS. The decision is left to the provider. Many local services routinely use WLS for all transports.

ALS providers were more likely to use WLS for transport than were BLS providers. This may relate to a sampling bias. ALS providers represented 75% of respondents. There was no stratification of the responses by role in the EMS system. BLS providers are more likely to work on non-emergency transfer ambulances, though one of the services involved in the survey provides BLS 911 service to a small city. ALS providers may fill either the 911 or the transfer role, but some bias may be introduced in that the patients transported by ALS crews are more likely to be critical and require more interventions, increasing the likelihood that WLS would be used.

A surprisingly high number of providers surveyed had been involved in ambulance collisions in the past. Previously experiencing an ambulance collision had some influence on responses to the statements on the survey. Only the extreme ranges of responses demonstrated statistical significance.

A visual inspection of the data displayed in Figure 3b suggests that the surveyed providers lack a strong consensus as to whether WLS use improves patient outcomes, though a trend toward disagreement is noted. Published data is mixed on this point. Some papers suggest an increase in mortality for trauma associated with increased out-of-hospital time.^15^,^22^ Other research points to a lack of benefit for trauma^16^,^17^ and other conditions.^19^,^23^,^24^ As more diagnoses are managed with scrutiny of associated time metrics (eg. ST-elevation myocardial infarction and acute stroke), a sense of time pressure may be felt by the providers, which may be a contribution to the responses.

A visual analysis of the data displayed in Figure 3a reveals a tendency toward agreement with the concept that WLS use shortens transport times. The published literature agrees with this response, reflecting a small but consistent shortening of transport times under WLS conditions.^10^–^14^

EMS providers must practice within the boundaries set by state law and treatment protocols. Occasionally state laws address the safety risk of lights and siren in general terms. For example; Massachusetts General Law does not specifically address the use of WLS on ambulances, but addresses the rights of ambulances to violate traffic regulations, which implies the use of WLS. This right is limited in MGL Chapter 89; 7B to use “in an emergency,” only with the application of due regard for the safety of the patient and the public.^25^

Some states have adopted regulations and protocols for the limitation of transportation with WLS. The Commonwealth of Pennsylvania, for example, instituted regulations and statewide treatment protocols which limited the use of WLS to medically necessary situations.^26^ The Commonwealth of Pennsylvania further codifies the specific operation of emergency vehicles, imposing an increase in regulation and promoting the safe operation of emergency vehicles.^27^ There remains, however, no national consensus on how to address the use of WLS during transport.

Massachusetts Motor Vehicle Regulations are broad in their permission of use of WLS for ambulances, limiting the criteria to “in an emergency” without further specification.^25^ In an addition to the protocols that was not present at the time the survey was conducted, Massachusetts Statewide Treatment Protocols address the use of WLS in a single sentence in the Routine Care Protocol:

Use of lights and sirens should be justified by the need for immediate medical intervention that is beyond the capabilities of the ambulance crew using available supplies and equipment.^28^

The lack of more specific regulation may contribute a sense of freedom to use WLS at will. When discussing the rate of WLS use with providers, providers commonly argue that the emergency is determined by the fact that 911 was called or by the patient’s perception of emergency. Anecdotally, the authors have found that a common explanation from local providers for use of WLS for otherwise minor complaints is the need to return the ambulance to service and thereby reduce the need for a mutual aid service to cover calls. The evidence that the time saved in these transports is an average of 3–4 minutes vacates this argument.

Survey responses to statement 4 differed significantly between those who had previously experienced an ambulance collision and those who had not. This raises questions as to whether having been involved in a collision begins to affect the belief in the need for WLS in order to satisfy service needs as opposed to patient-centered needs.

## LIMITATIONS

This is a limited data set representing a small fraction of prehospital providers. The data collected are not stratified by EMS system role, which may introduce bias in the amount of WLS used. The data span the areas of urban and suburban, but exclude true rural areas. We collected data from hospital-based, fire-based, and private EMS services, but did not include volunteer services. Finally, the size of the dataset and the scales used on the survey prevented a robust statistical analysis of the results, limiting some outcomes to inferences based on visual analysis.

## CONCLUSION

The data demonstrate the surveyed providers are aware of the risk posed by WLS to themselves, their patients, and the public. Nevertheless, their practice in the absence of rigid protocols suggests they disregard this knowledge. Despite a large number of prior ambulance collisions among the surveyed group, a high number of transports are conducted using WLS.

Further education needs to be conducted among providers to increase their knowledge of the published data. More focused research into providers’ motivations for use of WLS in the face of evidence of risk and questionable benefit may help guide education efforts in the future. Protocol and regulatory changes should be implemented to limit the use of WLS to those few patients who are most likely to derive a benefit.

## Figures and Tables

**Table 1:** Characteristics of emergency medical services responders to a survey on the use of lights and siren.

Characteristic	Mean (95% CI)
Age (years)	35 (33–37)
Minimum age	21
Maximum age	67
Gender, *n* (%)	
Total surveys completing this response	107
Female	24 (22)
Male	83 (78)
Experience (years)	
Total (*n*=106)	13 (11–15)
ALS providers (*n*=79)	
Total experience	14 (13–16)
ALS experience	9 (8–11)
BLS providers (*n*=27)	
Total experience	9 (5–13)
Estimated % WLS transports	
All providers	82 (77–87)
ALS providers	89 (84–94)
BLS providers	61 (50–73
Collisions, *n*, (%)	
Providers involved in collisions	59 (55)
Providers involved in >1 collision	34 (32)

*ALS*, advanced life support; *BLS*, basic life support *WLS*, warning lights and sirens

**Table 2:** Collisions reported by emergency medical service providers.

	N
Total collisions reported	147
Median collisions per provider, *n* (range)	1 (0–12)
Providers involved in collisions, *n* (%)	59 (55)
Providers involved in >1 collision, *n* (%)	34 (32)
Collisions using WLS, *n* (%)	59 (40)
Collisions per year of service in EMS	0.1

*WLS*, warning lights and sirens; *EMS*, emergency medical services
